# Differential impact of cognitive computing augmented by real world evidence on novice and expert oncologists

**DOI:** 10.1002/cam4.2548

**Published:** 2019-09-11

**Authors:** Donna M. McNamara, Stuart L. Goldberg, Lisa Latts, Deena M. Atieh Graham, Stanley E. Waintraub, Andrew D. Norden, Cody Landstrom, Andrew L. Pecora, John Hervey, Eric V. Schultz, Ching-Kun Wang, Nicholas Jungbluth, Phillip M. Francis, Jane L. Snowdon

**Affiliations:** ^1^ Division of Breast Oncology John Theurer Cancer Center at Hackensack University Medical Center Hackensack NJ USA; ^2^ Cota Inc. New York NY USA; ^3^ IBM Watson Health Cambridge MA USA

**Keywords:** artificial intelligence, electronic health records, point‐of‐care systems, real world evidence

## Abstract

**Introduction:**

Cognitive computing point‐of‐care decision support tools which ingest patient attributes from electronic health records and display treatment options based on expert training and medical literature, supplemented by real world evidence (RWE), might prove useful to expert and novice oncologists. The concordance of augmented intelligence systems with best medical practices and potential influences on physician behavior remain unknown.

**Methods:**

Electronic health records from 88 breast cancer patients evaluated at a USA tertiary care center were presented to subspecialist experts and oncologists focusing on other disease states with and without reviewing the IBM Watson for Oncology with Cota RWE platform.

**Results:**

The cognitive computing “recommended” option was concordant with selection by breast cancer experts in 78.5% and “for consideration” option was selected in 9.4%, yielding agreements in 87.9%. Fifty‐nine percent of non‐concordant responses were generated from 8% of cases. In the Cota observational database 69.3% of matched controls were treated with “recommended,” 11.4% “for consideration”, and 19.3% “not recommended.” Without guidance from Watson for Oncology (WfO)/Cota RWE, novice oncologists chose 75.5% recommended/for consideration treatments which improved to 95.3% with WfO/Cota RWE. The novices were more likely than experts to choose a non‐recommended option (*P* < .01) without WfO/Cota RWE and changed decisions in 39% cases.

**Conclusions:**

Watson for Oncology with Cota RWE options were largely concordant with disease expert judged best oncology practices, and was able to improve treatment decisions among breast cancer novices. The observation that nearly a fifth of patients with similar disease characteristics received non‐recommended options in a real world database highlights a need for decision support.

## Graphical abstract

Cognitive computing point‐of‐care decision support tools which ingest patient attributes from electronic health records and display treatment options based on expert training and medical literature, supplemented by real world evidence, might prove useful to expert and novice oncologists. We compared recommendations from one platform and noted concordance with disease expert opinions. The platform was also able to influence novice oncologists in test cases. Since nearly a fifth of similar real world cases received non‐recommended therapies. Our study demonstrates a need for decision support tools.

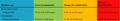

## 1 INTRODUCTION

The practice of oncology has become increasingly complex as a result of rapid expansion in the scientific knowledge of cancer biology, new therapeutic approaches, and an increased emphasis on value. The oncologist looking for guidance in treatment selection may need to search through scores of medical journals, national treatment guidelines (such as those published by the National Comprehensive Cancer Network), “paid” recommendation websites (such as UptoDate and OncoDoc2), primary literature websites (such as PubMed and Google Scholar), research and clinical trial websites (such as http://ClinicalTrials.gov), and institutional or payer‐driven pathways programs.^1^, ^2^, ^3^, ^4^, ^5^ This explosion of biomedical information may make it difficult to find relevant information for a particular patient during a busy clinic schedule. Point‐of‐care clinical decision support systems, available in the clinic at the time of treatment selection, that synthesize this diverse medical information, while accounting for unique patient factors and correcting for local capabilities, might therefore prove useful in routine cancer management.^6^, ^7^, ^8^, ^9^

IBM Watson for Oncology (WfO) is a cognitive computing point‐of‐care system that provides confidence‐ranked, evidence‐based treatment options for patients with cancer. Unlike simple computerized search and retrieve programs that are explicitly programmed, cognitive computing refers to systems that learn at scale, reason with purpose, and interact with humans naturally based on communication (eg voice) and training. Watson for Oncology processes structured and unstructured data from the medical literature, treatment guidelines, medical records, imaging, lab and pathology reports, and is guided by expert Memorial Sloan Kettering Cancer Center (MSKCC, New York, NY) training to formulate therapeutic options. To develop breast cancer treatment options WfO reviews more than 300 medical journals and textbooks supplemented with additional literature and guidelines chosen by MSKCC. Watson for Oncology was initially trained using an iterative process involving over 550 test breast cancer cases, with WfO options reviewed and scored by experts at MSKCC, and the feedback incorporated into the cognitive learning system. The evidence‐based supported treatment options are presented to the clinician in three categories: “recommended”, representing the MSKCC preferred approach; “for consideration”, evidence‐based alternative treatments; and “not recommended”, alternative therapies that may be unacceptable; along with supporting literature and toxicity profiles based on clinical trial data.^10^

Since patients treated in routine clinical practice may not have the same characteristics as research subjects enrolled in clinical trials, presenting “real world” treatment patterns and outcomes as a component of decision support systems may enhance recommendations. Benchmarks drawn from patients with similar characteristics treated at the same center or elsewhere in the country can provide rationale for practice transformation. The WfO platform is currently being augmented to display real world evidence (RWE) provided by Cota Inc. Cota draws patient demographic, clinical, treatment and outcome data from the electronic health records of providers within a national observational database. The Cota RWE platform utilizes a precise classification schema called the Cota Nodal Address (CNA) to identify patients with similar characteristics based on relevant patient and disease prognostic elements.^11^ The combined WfO‐Cota RWE point‐of‐care solution provides the oncologist with both the WfO options and the Cota historical real‐world treatment selections including 1‐ and 3‐year survival outcomes for patients with similar characteristics treated at the oncologist's cancer center and within the entire national database.

Confirmation that the treatment options from a cognitive computing system are concordant with human expert opinions would be reassuring to clinicians. Comparison studies of WfO against recommendations at a tumor board in India noted concordance in 93% of breast, 96.4% of lung, 81.0% of colon and 92.7% of rectal cancer cases.^12^, ^13^ A review in Thailand noted concordance rates of 89% for colorectal, 91% for lung, 76% for breast, and 78% for gastric cancer, with variance often driven by regional guidelines.^14^ An expansion of the 2016 Thailand study conducted in 2017 for a total of 590 cases found the overall concordance rate for the retrospective cases was 81%; 78% for bladder, 70% for breast, 56% for cervical, 90% for colon, 82% for gastric, 93% for lung, 79% for ovarian, 82% for prostate and 94% for rectal. Similarly, the overall concordance rate for the prospective cases was 80%; 75% for bladder, 72% for breast, 78% for cervical, 95% for colon, 71% for gastric, 87% for lung, 63% for ovarian, 86% for prostate and 76% for rectal cancers.^15^ A review in China noted recommendations were concordant in 79.0% of breast cancers and 96.9% in lung cancers, with the main reason for disagreement being economic constraints.^16^, ^17^ A recent review in China for Stage II colon cancer patients found overall concordance was 89.1% with high‐risk subgroup results ranging from 87.5% (*P* = .68) in T4 primary to 92.7% (*P* = .02) in poorly differentiated histology.^18^ Another Chinese report demonstrated concordance between IBM Watson for Oncology and a multi‐disciplinary tumor board among 79% early stage and 70% metastatic breast cancer patients.^19^ A lower concordance was noted in Korea among patients with colon cancer, attributed largely to age, reimbursement plan, omitting chemotherapy after liver resection, and not recommending biologic agents.^20^ Another Chinese review noted increasing discordance among older breast cancer patients.^21^

Similar confirmations of concordance at USA centers of excellence have not been reported. The influence of the WfO options on subsequent treatment decisions, comparisons with real world treatments (a potential surrogate to explore need for decision support) and the ability of the system to improve treatment strategies by oncologists who are not experts in a specific cancer has not been explored.

## 2 METHODS

Case descriptions of 88 postmenopausal women with breast cancer evaluated at a referral cancer center (John Theurer Cancer Center, Hackensack, NJ, USA) between August 2017 and April 2018 were prepared. The cases were drawn at random from the electronic health record at the center (Epic). The descriptions included clinical characteristics including gender, age, menopausal status, and performance status; descriptions of prior therapies for early stage cancer including if surgery was performed, type of surgery, and lymph node evaluation procedures; staging procedures including tumor size (clinical and pathologic, if applicable), number of involved lymph nodes (clinical and pathologic determinations including micro‐metastasis, if applicable), metastatic status (sites if applicable), and summation stage; surgical findings and margins; primary tumor findings including histology, tumor grade, estrogen and progesterone receptor status, her2neu oncogene status including testing method, presence of lympho‐vascular invasion, and OncotypeDx recurrence scores; key laboratories including liver associated enzymes; pertinent co‐morbidities; and genetic characteristics including brca‐1 and brca‐2 mutational status. Not all characteristics were available for all cases as documented in the actual electronic health records.

The cases were shown to three oncologists who specialize in the care of breast cancer at the referral center. The physicians were asked to record their recommended treatment strategies for each case including choice of chemotherapy and/or hormonal agent and whether referral for radiation therapy was appropriate. The physicians were subsequently shown the options for treatment based on the WfO platform for a random selection of 25‐50 cases and then asked to again indicate their preferred treatment strategy.

A selection of 25‐50 cases were shown to 4 oncologists at the John Theurer Cancer Center who primarily cared for patients with solid tumors other than breast cancer (1 each gastroenterology, genitourinary, general oncology and lung cancer) and to 6 physicians who cared for patients with hematologic malignancies. These physicians rendered their treatment strategies without and then with assistance from the WfO platform.

To determine actual real world treatment patterns for patients with similar characteristics the attributes for the 88 cases were entered into the Cota RWE platform. For each case the system was queried to identify patients with similar attributes (matched using the CNA system) and the most common historical treatment in the national database was identified.

## 3 RESULTS

### 3.1 Concordance of WfO treatment options with Disease Specific Expert Opinions

The breast cancer experts reviewed 223 cases in total (not all cases scored by each physician). Without assistance the WfO “recommended” option was concordant with selection by breast cancer experts in 175 (78.5%) cases. A “for consideration” option was selected in 21 (9.4%) cases. Overall, an acceptable WfO treatment strategy was chosen in 87.9% cases. A “not recommended” by WfO treatment was selected by the breast cancer experts in 27 cases (12%; Table 1). Seven of the 88 cases (8%) generated 59% of non‐concordant responses with ≥2 doctors disagreeing with WfO (Table 2). One of the three breast cancer experts had previously practiced at Memorial Sloan Kettering Cancer Center (5 years prior to the study). Interestingly, she deviated the least from MSKCC trained WfO platform suggesting a possible center effect predisposition in the options (84% “recommended” vs 75%; *P* = .1).

**Table 1:** Concordance of WfO options with breast cancer experts and actual real world historical treatment selections

Physician type	Green (recommended)	Orange (for consideration)	Red (not recommended)
Breast cancer expert			
Without WfO	78.5% (175 of 223 cases)	9.4% (21 of 223 cases)	12.1% (27 of 223 cases)
Cota database			
Historical treatment	69.3% (61 of 88 cases)	11.4% (10 of 88 cases)	19.3% (17 of 88 cases)

Abbreviation: WfO, Watson for Oncology.

**Table 2:** Discordant cases (≥2 of the 3 breast cancer experts)

Case	WfO	Experts	Key characteristics
1	CMF	ACT, ACT, TC	66 y, stage IA, pT1cN0, ductal, high grade, ER/PR+, her2−, oncotype high
2	Hormonal	CMF, CMF, CMF	85 y, stage IIIA, pT2N2a, 5 + LN, ductal, high grade, ER/PR+, her2−, lym‐vasc inv+
3	CMF	Hormonal, hormonal	77 y, stage IA, pT1bN0, mix histology, inter grade, ER+, PR−, her2−, oncotype intermed
4	CMF, ACT	Hormonal, TC	57 y, stage IIA, T2N0, ductal, interm grade, ER/PR+, her2−, oncotype inter, lym‐vasc+
5	ACT	Hormonal, CMF	73 y, stage IIA, pT1mi, 1 LN (2 mm), ductal, interm grade, ER/PR−, her2−, lym‐vasc−
6	ACT	Hormonal, CMF	75 y, stage IA, pT1cN0, ductal, high grade, ER/PR/her2−, lym‐vasc−
7	Hormonal	ACTH, TH	64 y, recurrent chest wall with movable LN, prior AC 36 mo, ER/PR/her2+

Abbreviation: ACT: doxorubicin, cyclophosphamide, docetaxel; ACTH: doxorubicin, cyclophosphamide, paclitaxel, trastuzumab; CMF: cyclophosphamide, methotrexate, 5‐fluorouracil; TC: docetaxel, cyclophosphamide; TH: paclitaxel, trastuzumab; WfO, Watson for Oncology.

### 3.2 Comparison of WfO treatment options with Historical Real World Treatments

Watson for Oncology with Cota RWE point of care displays the most common utilized regimens and the 1‐ and 3‐year overall survival rates for patients with similar characteristics to provide point‐of‐care decision support. The Cota RWE database (cutoff May 9, 2018) contained detailed demographic, clinical, treatment and outcome information on 14 917 patients with breast cancer. These patients were evaluated at 30 USA cancer centers by 214 oncologists. For each test case the key characteristics were matched to generate a cohort of similar patients for comparison and the most commonly utilized treatment strategy in the national database was recorded. Using the Cota national database 69.3% of breast cancer patients with similar characteristics (matched historical controls) were most commonly treated with WfO “recommended” treatment strategies. A “for consideration” strategy was the most commonly utilized treatment choice in 11.4%. However, in 19.3% cases a “not recommended” regimen was the most common treatment strategy (Table 1). Thus the historical patients, treated in both academic and non‐academic centers, had a trend towards more non‐recommended therapies compared with the breast cancer experts at the John Theurer Cancer Center. (*P* = .1).

### 3.3 Influence of WfO with Cota RWE on decision‐making

A subset of cases was shown to oncologists with varying levels of expertise in the care of patients with breast cancer. Without guidance from WfO breast cancer subspecialists chose 89.7% recommended/for consideration treatments which did not significantly change (88.5%) with subsequent assistance by the WfO platform. By contrast oncologists who were not routinely caring for patients with breast cancer appeared to benefit from the guidance from WfO. Without reviewing WfO, the breast cancer novice oncologists chose 75.5% recommended/for consideration treatment options which improved to 95.3% with WfO. Breast cancer novices were more likely than experts to choose a non‐recommended option (*P* < .01) without WfO, but with decision support they were more consistent with WfO top options (*P* = .03). Hematologic malignancy physicians obtained greatest insights (22% changes). Breast cancer experts assisted by WfO changed preferred treatment decisions 6.4% of the time while solid tumor and hematologic focused oncologists changed decisions 39.0% and 39.3%, respectively (Table 3).

**Table 3:** Selection of recommended treatment options with and without WfO decision support by breast cancer experts and novice oncologists

Physician type	Green (recommended)	Orange (for consideration)	Red (not recommended)
Breast cancer expert			
Without WfO/Cota	80.8% (63 of 78 cases)	9.0% (7 of 78 cases)	10.3% (8 of 78 cases)
With WfO/Cota	80.8% (63 of 78 cases)	7.7% (6 of 78 cases)	11.5% (9 of 78 cases)
Solid tumor			
Without WfO/Cota	66.7% (70 of 105 cases)	8.6% (9 of 105 cases)	24.8% (26 of 105 cases)
With	86.7% (91 of 105 cases)	4.8% (5 of 105 cases)	8.6% (9 of 105 cases)
Heme malignancy			
Without WfO/Cota	60.7% (142 of 234 cases)	15.0% (35 of 234 cases)	24.4% (57 of 234 cases)
With	88.5% (207 of 234 cases)	8.6% (20 of 234 cases)	3.0% (7 of 234 cases)
BC novices (solid + heme)			
Without WfO/Cota	62.5% (212 of 339 cases)	13.0% (44 of 339 cases)	24.5% (83 of 339 cases)
With	87.9% (298 of 339 cases)	7.4% (25 of 339 cases)	4.7% (16 of 339 cases)

## 4 CONCLUSIONS

This study, similar to those conducted in Asia, demonstrates that the cognitive computer platform IBM WfO is able to render cancer treatment options in concordance with expert opinions.^12^, ^13^, ^14^, ^15^, ^16^, ^17^, ^18^, ^19^, ^20^, ^21^ Specifically, in this USA based study 3 blinded oncologists who specialize in the care of breast cancer patients chose treatment strategies in agreement with WfO acceptable options 87.9% of the time, and chose a preferred option in 78.5% of cases. The oncologist who had previously worked at MSKCC more often agreed with the MSKCC trained WfO system suggesting a possible center effect. The difference was not statistically significant, likely as a result of limited sample size. A small number of cases (8%) generated the majority of disagreements between WfO and breast cancer expert recommendations. In these cases, presenting the WfO platform outputs failed to change treatment choices, suggesting either areas where WfO needs additional training or meaningful institutional biases. During pilot testing of the system at the John Theurer Cancer Center it was noted by the oncologists that the options for older women by WfO were more aggressive than local institutional preferences (data not shown).

The true value of a cognitive computing application however is not to confirm that experts choose appropriate therapies. Instead, a decision support tool needs to improve care when delivered by novices. As oncology becomes more complex it may become harder for the “generalist” oncologist to keep abreast of subtle nuances that affect treatment selections across a wide range of tumor types. Prior studies have suggested that computerized decision support systems can improve practitioner performance.^22^, ^23^ In our study, oncologists who do not routinely care for breast cancer patients chose WfO “recommended” options only 75.5% of the time. However, with guidance by the WfO platform the novices reconsidered their treatment selection and subsequently chose recommended options 95.3% of the time. These findings are in alignment with a recent study evaluating 619 healthcare providers who entered 1018 metastatic breast cancer test cases into an online tool. Among participating oncologists whose initial intended treatment of metastatic breast cancer differed from the experts, 51% indicated that they would change their choice of therapy.^24^

Although our study found that cognitive computing decision support tools may facilitate physician choices in care, especially by novice oncologists, acceptance by patients of computerized recommendations needs to further explored. A series of patient focus groups conducted by MSKCC investigators, who helped train WfO, suggests high levels of interest, perceived value, and acceptance of computerized support, as long as the tools is used to supplement and not replace physician decision making. Participants also described important concerns, including the need for strict processes to guarantee the integrity and completeness of the data presented and the possibility of physician overreliance on computerized recommendations.^25^

A comparison of the WfO options with actual treatments delivered in real world settings as noted by the Cota RWE observational database demonstrates a need for clinical decision support. In the historical real‐world database 19.3% of patients with characteristics similar to the test cases received therapies that were not recommended by WfO. Since approximately a third of the Cota database cases are drawn from tertiary cancer centers (with presumed availability of disease specific experts), the gap in treatment decisions might even be larger. As oncologic care increasingly relies on molecular characteristics, additional decision support may be needed.^26^ The companion solution, Watson for Genomics (WfG), which analyzes and categorizes genetic alterations that are related to cancer progression and suggests potential treatments, was able to identify actionable mutations in one‐third of patients compared to a university molecular tumor board.^27^ A recent WfG review in Spain found investigational options were identified in most cases from a variety of samples.^28^

There were several limitations of the study which might affect generalization of the findings. We focused on a small subset of breast cancer test cases, namely post‐menopausal earlier stage patients, in line with the most frequently identified in clinical practice. Adding a larger diversity of cases, including rare or more controversial/challenging scenarios, we suspect might increase the gap between evidence‐based WfO and clinician responses, lowering the concordance but increasing the value of decision support. Additionally, to conduct the study, physicians were not given the same time to ponder and research treatment strategies with either the WfO platform or using their own normal methods that they might utilize in clinical practice. However, real world studies have suggested that the average oncology visit is less than 30 minutes.^29^, ^30^ Thus the availability of a point‐of‐care decision support that synthesizes large amounts of bio‐information tailored to individual patient characteristics and delivers this instantaneously might be important to the busy clinician. In summary, this study suggests that WfO with Cota real word evidence generates options largely concordant with USA breast cancer experts and that these options may influence clinical care in real world scenarios, especially among non‐subspecialist oncologists.

## Data Availability

Data available on request from the authors.
